# SMS for Life: a pilot project to improve anti-malarial drug supply management in rural Tanzania using standard technology

**DOI:** 10.1186/1475-2875-9-298

**Published:** 2010-10-27

**Authors:** Jim Barrington, Olympia Wereko-Brobby, Peter Ward, Winfred Mwafongo, Seif Kungulwe

**Affiliations:** 1 SMS for Life Program Director, Forum 1.P-94, Novartis Campus, CH-4056 Basel, Switzerland; 2 Project Support, Forum 1.P-94, Novartis Campus, CH-4056 Basel, Switzerland; 3 IBM (UK) Ltd, MP9, PO Box 31, Birmingham Rd, Warwick, CV34 5JL, UK; 4 Senior Health Officer, National Malaria Control Program, Ministry of Health & Social Welfare, Ocean Road - NIMR Offices, Box 9083, Dar-es-Salaam, Tanzania; 5 District Medical Officer, Lindi District Council, P.O. Box 328, Lindi, Tanzania

## Abstract

**Background:**

Maintaining adequate supplies of anti-malarial medicines at the health facility level in rural sub-Saharan Africa is a major barrier to effective management of the disease. Lack of visibility of anti-malarial stock levels at the health facility level is an important contributor to this problem.

**Methods:**

A 21-week pilot study, 'SMS for Life', was undertaken during 2009-2010 in three districts of rural Tanzania, involving 129 health facilities. Undertaken through a collaborative partnership of public and private institutions, SMS for Life used mobile telephones, SMS messages and electronic mapping technology to facilitate provision of comprehensive and accurate stock counts from all health facilities to each district management team on a weekly basis. The system covered stocks of the four different dosage packs of artemether-lumefantrine (AL) and quinine injectable.

**Results:**

Stock count data was provided in 95% of cases, on average. A high response rate (≥ 93%) was maintained throughout the pilot. The error rate for composition of SMS responses averaged 7.5% throughout the study; almost all errors were corrected and messages re-sent. Data accuracy, based on surveillance visits to health facilities, was 94%. District stock reports were accessed on average once a day. The proportion of health facilities with no stock of one or more anti-malarial medicine (i.e. any of the four dosages of AL or quinine injectable) fell from 78% at week 1 to 26% at week 21. In Lindi Rural district, stock-outs were eliminated by week 8 with virtually no stock-outs thereafter. During the study, AL stocks increased by 64% and quinine stock increased 36% across the three districts.

**Conclusions:**

The SMS for Life pilot provided visibility of anti-malarial stock levels to support more efficient stock management using simple and widely available SMS technology, via a public-private partnership model that worked highly effectively. The SMS for Life system has the potential to alleviate restricted availability of anti-malarial drugs or other medicines in rural or under-resourced areas.

## Background

Artemisinin-based combination therapy (ACT) is recommended by WHO for first-line treatment for uncomplicated *Plasmodium falciparum *malaria [1], in recognition of the superior efficacy and faster symptomatic improvement observed with ACT compared to other treatments [2,3], as well as a reduction in gametocyte carriage among ACT-treated patients that could potentially contribute to a lower rate of disease transmission [1,4,5].

Maintaining adequate supplies of ACT at the health facility level in rural areas of sub-Saharan Africa, however, can be highly challenging. Poor supply chain management, including limited or non-existent stock control and forecasting, means that even though anti-malarial drugs may be available centrally there can be frequent stock-outs at the local level, which often last for extended periods. As a result, patients may have to travel long distances to obtain ACT or, all too often, remain untreated with the consequent risk of developing severe disease, organ damage and death.

Tanzania has the third largest population at risk of malaria, with 11 million cases of malaria occurring each year [6]. ACT represents first-line therapy in the country, although rapid diagnostic tests (RDT) are only used to confirm the diagnosis where health facilities have this resource; otherwise, the diagnosis is made on the basis of clinical symptoms. Anti-malarial therapies are distributed via one of two mechanisms in Tanzania. Products can be issued to health facilities automatically in fixed quantities on a quarterly basis, with requirements determined at district level by the District Medical Officer (DMO) and at national level by the National Malaria Control Programme (NMCP) (the 'push' system). Alternatively, they can be distributed every month in response to individual requests from health facilities that are sent by the DMO for approval by the Ministry of Health, after which medicines are dispatched via an Integrated Logistics System (ILS) (the 'pull' system). In both cases, medicines are stored and dispatched from one of nine Zonal Stores in the country.

Recognizing that standardly-available technology has the potential to improve supply management for anti-malarial medicines in rural regions, a collaborative partnership of public and private institutions was set up under the auspices of the Roll Back Malaria Partnership to undertake a 21-week pilot project in Tanzania. The objective of the project was to improve the supply, planning and access to ACT therapy through use of mobile telephones, SMS messages and electronic mapping technology. The results of this pilot project, 'SMS for Life', are reported here.

## Methods

### Objectives

The objectives of the SMS for Life pilot were three-fold: (1) to demonstrate that visibility of weekly stock levels of key anti-malarial medicines at the health facility level will promote action to eliminate and/or reduce stock-outs (2) to demonstrate that a state-of-the-art data gathering infrastructure can be made available via simple tools such as SMS and mobile telephones in remote locations in sub-Saharan Africa (3) to demonstrate the effectiveness of a public-private partnership model.

### Location

Of the 131 districts in Tanzania, three rural districts (Lindi Rural, Ulanga and Kigoma Rural) were selected by the NMCP for inclusion in the pilot, covering a total population of 1.2 million. The selected districts met all four criteria for inclusion. First, the districts were to differ in terms of level of health service delivery and access, with the aim of providing a broadly representative sample of the entire country. Lindi Rural is an 'average' district. Ulanga is a challenging district in terms of staff shortages, skill level and remote location. Kigoma Rural also presents problems, due to its large geographic size and long distances between the Zonal Store and remote health facilities. Second, the districts were all to be in different regions of the country, and supplied by different Zonal Stores. Third, all districts were to be malaria endemic with malaria the most common cause of death. Fourth, selected districts were not to be involved in other pilot projects.

Lindi Rural, Ulanga and Kigoma Rural districts included 48, 30 and 51 health facilities, respectively i.e. 129 health facilities in total. The Lindi Rural and Kigoma Rural districts operate anti-malarial supply using a 'pull' system via ILS. The Ulanga district is undergoing a transition from a 'push' system to the 'pull' system.

### Duration and scope of the SMS for Life pilot

The pilot study was 21 weeks in duration. This period was chosen because it covered two quarterly order cycles and five monthly delivery cycles. Data collection started on 1^st ^October 2009 and ended on 25^th ^February 2010.

The system covered stocks of artemether-lumefantrine (AL, Coartem^®^, Novartis Pharma AG, Basel, Switzerland) and injectable quinine (provided by multiple manufacturers). Stocks of four different dosage packs of AL were included: 'yellow' packs used for babies weighing 5 kg to < 15 kg, 'blue' packs for children weighing 15 kg to < 25 kg, 'red' packs for children weighing 25 kg to < 35 kg and 'green' packs for children weighing 35 kg or more and for adults.

### The SMS for Life system

The system consists of two components: an SMS management tool and a web-based reporting tool.

#### SMS management tool (Figure 1)

**Figure 1:**
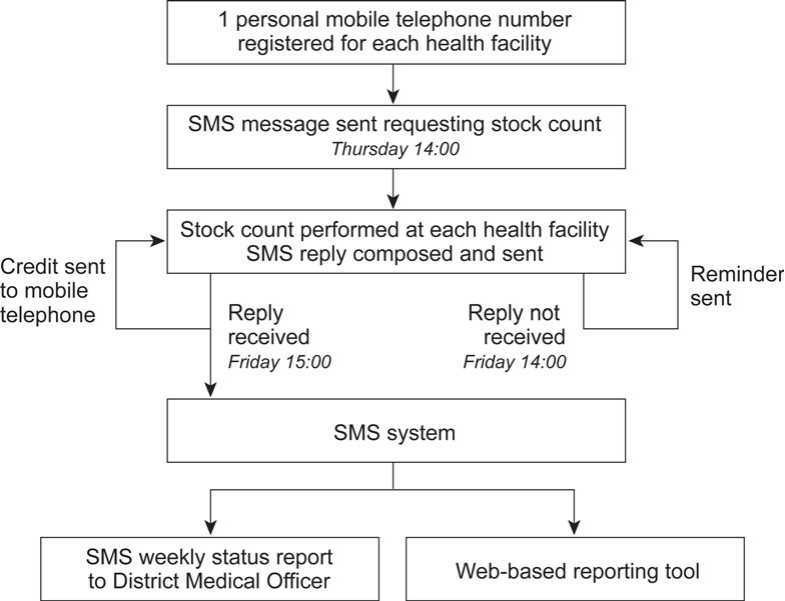
**Schematic of the SMS system in the SMS for Life pilot**.

The SMS application stores a single registered mobile telephone number for one healthcare worker at each health facility. Once a week, a stock request is sent by SMS to each of these telephone numbers. Stock messages are sent back in reply using a free short code number at zero cost to the healthcare worker i.e. telephones do not need to be in credit to reply. A standard message format is used to capture stock quantities of AL and quinine, and formatting errors are handled through follow-up SMS messages to the facility.

##### Step 1

A personal mobile telephone number for one healthcare worker at each health facility in the three pilot districts was obtained during training sessions and registered with the SMS application. Only stock count messages from registered personal mobile telephone numbers are accepted.

##### Step 2

Every Thursday at 14:00 an SMS message is sent to all registered health facility workers requesting stock counts.

##### Step 3

Full boxes of AL in the storeroom of each facility are counted, and individual quinine injectable vials are counted in the storeroom and dispensary (the difference in accounting methodologies was at the request of the NMCP).

##### Step 4

An SMS message is composed by the health facility worker, comprising a code for each type of medicine and the quantity, following an agreed format.

##### Step 5

The heath facility worker either replies to the stock request SMS or sends a new SMS using the free short code number. If the message is sent in an incorrect format, the system automatically informs the sender. After three unsuccessful attempts, the district management is informed and asked to intervene.

##### Step 6

The SMS system sends an automatic reminder to all health facilities that have not replied by Friday at 14:00.

##### Step 7

The SMS system credits the healthcare worker's mobile telephone with a fixed amount of money (1000-1500 TZS, depending on the district) for personal use if the stock count reply is received before 17:00 on Friday. Late SMS replies are accepted until 13:00 on the following Thursday, but no credit is applied to mobile telephones for late replies.

##### Step 8

The system provides a weekly status report to the DMO indicating (a) which health facilities did not provide a stock count and (b) which health facilities have a stock-out.

#### Web-based reporting tool

The data captured from the SMS stock count messages is made available via a secure website for which access requires a unique user identification and password. Access is provided to the DMO and his/her staff in each participating district, the relevant Regional Medical Officers and their staff, the project team, the NMCP and the Medical Stores Department including the Zonal Stores affiliated with each district. The website provides (a) current and historical data on AL and quinine injectable stock levels at the health facility and district level (b) Google mapping of district health facilities with stock levels overlays and stock-out alerts (c) SMS messaging statistics e.g. errors, received messages and (d) usage statistics.

### District-level management

The DMO appointed one person in the district to redistribute medicines in response to stock-outs identified by the SMS for Life system. Redistribution could be undertaken by telephoning health facilities with stock-outs to inform them of excess stock in a neighboring health facility, or by contacting the Malarial Focal Person in the district to request that they move stock from a health facility with a high stock level to a neighboring facility.

### Participant training

Training was provided at three levels:

(i) At a national level, core project and system training was provided at a half-day session for NMCP, Medical Stores Department and additional staff to explain the project objectives, use of the reporting system and action to be taken based on stock count information provided.

(ii) At the district level, a half-day training session was provided for the DMO, Malaria Focal Person, District Pharmacist and Zonal Store representative for each district. Training covered use of the reporting system, action to be taken based on stock count information provided, and education and assistance for health facility workers.

(iii) At the health facility level, a half-day training session was provided by the NMCP in-country project lead for health facility workers within each district, in the local language. The session included registration of personal mobile telephone numbers, the procedure for counting stock, composition of the SMS stock count messages, live simulations of counting, composing and sending SMS messages, and best practice for stock management and storage of anti-malarials.

### Monitoring and evaluation

Weekly stock reports, stock-out statistics, error rates, deliveries and system access were monitored daily online during the 21-week pilot study. Surveillance visits were undertaken for 116/129 health facilities (90.0%) at least once to validate the accuracy of stock count data provided by health facility workers.

District management team members were interviewed towards the end of the pilot study to assess stock movement during the study, obtain feedback on use and ease of access to the data system and on use of the registration/de-registration function for health facility mobile telephone numbers, seek views on training and training materials, and elicit opinions on the SMS for Life project versus other stock management practices and the potential for future implementation of the scheme. Throughout the project, information on every order and delivery of AL or quinine injectable from Zonal Stores was collected.

### Project partnership and contributions

The project partnership had a fixed-term commitment of less than one year, with no centralized budget, formal contract or memorandum of understanding. The Tanzanian Ministry of Health and Social Welfare, The Roll Back Malaria Partnership, Novartis Pharma AG, Vodafone and IBM took part in the pilot project. Each partner funded their own activities.

The NMCP in Tanzania, operating as part of the Ministry of Health and Social Welfare, was the owner and main user of the SMS for Life pilot and coordinated all project activities in the country i.e. planning, implementation and evaluation, including provision of a project leader and vehicles with drivers. The Roll Back Malaria Partnership provided project oversight, including the work of the steering committee, and led advocacy activities. Novartis initiated and led the pilot, defining the solution, sourcing partners, establishing the steering committee, and providing the necessary resources and funding (e.g. to support health professional training). Vodafone and its partner, Matssoft, supported the design, funding and development of the system application and the implementation of the technical solution, and funded all technical operational costs of the pilot. IBM supplied management resource support to the project and provided an on-line collaboration tool 'Lotus Live', which allowed all the project partners to coordinate their inputs across company networks.

## Results

### Data collection

**Figure 2:**
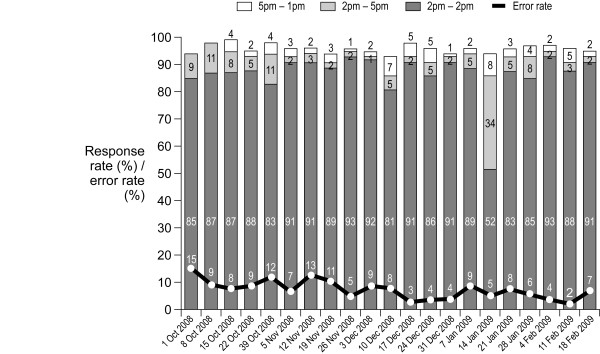
**Proportion of health facilities responding to SMS requests for stock counts according to timing of response, and error rate in responses, during the 21-week SMS for Life pilot**.

During the 21-week study, the average response rate to SMS requests for stock count data was 95%. The response rate did not fall below 93% at any point (Figure 2). The proportion of late replies (i.e. after 17:00 on Friday) was low, averaging 3% overall. The rate of responses, and the proportion of late responses, did not vary markedly during the pilot, other than after the request sent on 14^th ^January 2010 when there was a national problem with connectivity on one mobile telephone network (Figure 2). The highest response rate was in Lindi Rural (99%), compared to 93% in Ulanga and 94% in Kigoma Rural, which is likely to have been the result of disciplinary action in the Lindi Rural district, consisting of warning letters and interviews at the district office for non-compliant health facility staff. Across all three districts, feedback from district management and data from questionnaires completed by health facility workers indicated that the financial incentive of airtime credit was an important contributor to the high response rate.

The error rate for composition of SMS responses was low, averaging 7.5% throughout the study (Figure 2). In Lindi Rural, 100% of error SMS responses were corrected, and although data on corrected rate were not routinely collected the fact that the accepted response rate did not fall below 93% at any point confirms that even incorrect messages from the other two districts were usually corrected.

Stock counting, as assessed by surveillance visits to 116 of the 129 health facilities in the three districts, showed a data accuracy of 94% i.e. the most recent stock message matched the inventory inspected at the health facility.

### System usage

The central NMCP log-in was activated on average once a day. The central Medical Stores Department and the Zonal Stores in the three districts virtually never accessed the system. At the district level, the weekly emails sent by the SMS for Life system were read by at least one team member in the district management team of each district every week during the pilot, with the exception of a single email to the Kigoma team. System usage in the Lindi Rural district decreased as stock-outs were eliminated after week 8, declining from 45 log-ins during October 2009 to 13 log-ins during February 2010. In Ulanga, log-ins increased (35 log-ins during October-December, rising to 70 log-ins during the last 6 weeks of the project) after the Clinical Officer in the District Medical Office was given a Blackberry and more prescriptive input from the SMS for Life project team. In the third district, Kigoma Rural, access to the system was low in the early phase (33 times in October-December) but increased to 28 times in the last 6 weeks after the District Pharmacist and Malaria Focal Person were each given a Blackberrry device to access stock count data.

### Anti-malarial stock levels

**Figure 3:**
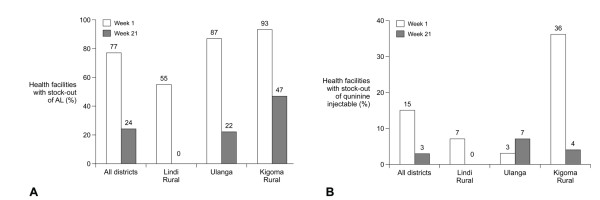
**Proportion of health facilities with stock-out of (a) 1 type of dosage pack of artemether-lumefantrine (AL) or (b) quinine injectable at the start (week 1) or end (week 21) of the SMS for Life pilot overall and by district**.

**Figure 4:**
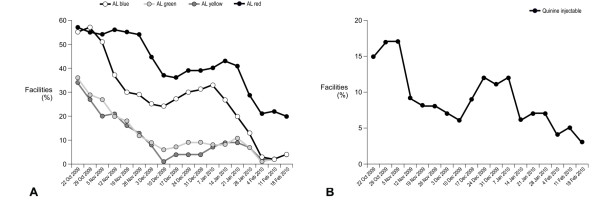
**Proportion of health facilities with stock-out of (a) each dosage pack of artemether-lumefantrine and (b) vials of quinine injectable during the SMS for Life pilot. Combined data from all three districts are shown**.

At the start of the pilot (week 1), 78% of health facilities had no stock of one or more of the four different AL dosage packs or of quinine injectable. By the end of the pilot (week 21), this proportion had fallen to 26%. The reduction in stock-outs was largely related to improvements in stocks of AL, since the proportion of health facilities with stock-outs of quinine at the start of the study was lower (18% compared to 77% of facilities with a stock-out of AL) (Figure 3). Stock-outs of all dosages of AL showed a progressive decline over the first two months of the pilot, with a gradual increase from the middle of December to the second half of January, reflecting the ILS delivery schedule. By the end of the pilot, stocks-out of AL blue, green and yellow were almost eradicated but a fifth of health facilities still had no AL red, almost entirely due to continuing stock-outs in the Kigoma Rural district (Figure 4a). Over 80% of facilities held stocks of quinine injectable at baseline, which increased to more than 95% by the end of the pilot (Figure 4b).

**Figure 5:**
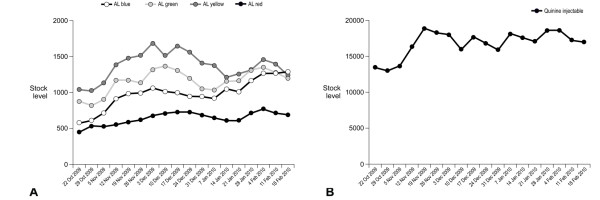
**Stock counts for (a) boxes of each dosage pack of artemether-lumefantrine and (b) vials of quinine injectable during the SMS for Life pilot. Combined data from all three districts are shown**.

Over the same period, total AL stock across the three districts increased by 64% from 2,696 boxes at week 1 to 4,411 boxes at week 21, while the number of quinine vials increased by 36% from 12,536 to 16,981 (36%). Stock levels showed a small increase for all AL dosages by week 21, with similar levels of AL blue, green and yellow, but stocks of AL red remained lower than for other dosages, again primarily due to the Kigoma Rural district (Figure 5a). Quinine injectable stock levels also showed a small increase during the pilot (Figure 5b).

There were marked differences between the three districts in terms of achievement of full stocking and in stock levels, for a variety of reasons. The Lindi Rural district was the most successful in managing stock levels, eliminating stock-outs for all five categories of medicine by week 8 and maintaining stocks of all three anti-malarials at almost all health facilities thereafter. Two key factors contributed. First, after receiving the first set of stock count data, the district management team made an emergency order to the Zonal Store. This delivery was distributed to health facilities according to priority based on their urgency of demand during weeks 2, 3 and 4, thereby eradicating most stock-outs. Second, when a health facility reported having only one box of any AL dosage pack, the district pharmacist either issued further stock or moved stock from a neighboring health facility in a pre-emptive manner. In the Ulanga district, the rate of stock-outs at week 1 was high (87% of health facilities), largely because no blue dosage packs of AL had been delivered to the district for almost a year. Also, Ulanga was transitioning from the 'push' system to ILS delivery during the pilot. As a result, deliveries were delayed and there were discrepancies between stock orders and the item delivered, for example with no blue AL dosage packs included and only very small quantities of other AL dosages. Furthermore, an emergency delivery was not received. Following two ILS deliveries, the second of which included blue AL dosage packs, 78% of all health facilities in Ulanga became fully stocked by week 21. The proportion of health facilities with no quinine injectable, however, increased from 3% at week 1 to 7% at week 21. In the third district, Kigoma Rural, almost all health facilities (93%) had a stock-out of at least one type of anti-malarial at week 1, and 36% were out of stock of all five products. There was an ongoing shortage of red AL dosage packs until the end of January 2010, with 42% of health facilities still having no red packs at the end of the study. Over 90% of health facilities, however, had stocks of all other products by week 21. The district relied only on regular ILS deliveries. Following the two ILS deliveries that were received during the pilot, the district management took 3-4 weeks to distribute medicines from the first delivery to all health facilities and after delivery of a complete ILS order in late December, including red AL dosage packs, stock counts of red packs only rose from 21 January onwards. Several factors contributed to outcomes in the Kigoma Rural district. The ILS delivery quantity for red dosage packs of AL was only sufficient to prevent stock-outs for three weeks, such that stock-outs were inevitable. Second, when red dosage packs were delivered they were distributed unevenly between health facilities, with some facilities receiving none, and no active redistribution was undertaken subsequently. Lastly, no emergency orders were submitted from the Kigoma Rural district despite severe stock shortages for the majority of the pilot.

## Discussion

The SMS for Life pilot achieved all three of its objectives. First, visibility of anti-malarial stock levels at the health facility level supported more efficient stock management. Across all three districts, the proportion of health facilities fully stocked with all five anti-malarial products increased from approximately one quarter to three quarters over the 21-week pilot. Second, the SMS for Life system brought accurate stock level information to all relevant parties using simple and widely available SMS technology that was easily accessed by appropriate users. Thirdly, the public-private partnership model worked highly effectively, and proved to be a major contributor to the success of the project.

To achieve full stocking of all five anti-malarial products required both an adequate starting level of products across the district and proactive redistribution of products by district management between health facilities. Redistribution is always likely to be required to compensate for delivery of varying quantities to different health facilities and varying consumption rates, particularly when there is a shortage of stock. By providing visibility of stock levels, the SMS for Life system meant that both of these criteria could be met, as demonstrated in the Lindi Rural district where health facilities were virtually all fully stocked after week 8 of the pilot. Comprehensive stock information was provided from health facilities, with an average response rate of 95%. Stock level information was accessible even in the remotest areas, and was provided via both weekly emails and secure web-based data to maximize usage. All aspects of the system proved easy to use after only a short training session. It was important to track log-ins by district staff and intervene as necessary by offering further training or additional access solutions (e.g. provision of Blackberry devices or computer modems); such interventions prompted dramatic increases in log-in rates in both the Ulanga and Kigoma Rural districts. By tracking weekly usage of all malaria products (ACTs, quinine and RCTs if used) by individual health facility, the system can profile annual requirements by facility, to inform and improve the accuracy of ordering and supply chain efficiency. From weekly usage of RDT's and ACT's the system can also calculate a proxy for the number of positive versus negative tests. While expiry dates were not tracked, a significant finding was that weekly visibility by facility led to DMOs being extremely active in implementing ongoing re-distribution of stock between facilities, thus reducing the risk of stock going out of date.

The pilot was implemented through a novel public-private partnership under the umbrella of the Roll Back Malaria Partnership. The SMS for Life solution was designed, built and implemented in less than a year, with no formal budget or legal contracts between partners. With a short timeframe and no ongoing financial commitments, this model was appealing to potential commercial partners, without whom the pilot could not have been undertaken.

A number of critical success factors were identified (Table 1). Government commitment at a high level is essential to ensure the system is workable and sustainable, and that its use is mandatory. Mobile telephone coverage within an acceptable distance (maximum 2-3 hours' walk from the health facility, although a period of no more than 15-30 minutes would be ideal) is a necessary prerequisite to participation. It is also crucial for health workers to use their personal mobile telephones, with which they are familiar and for which maintenance is not the responsibility of the project. Accordingly, a free number for sending stock information is mandatory since messages can still be sent if the telephone has no credit, a situation that can arise frequently. Although the pilot did not include a control arm without a financial incentive, feedback from health workers, district management and the NMCP indicated that a credit incentive for timely responses was key to the high response rates observed. The training sessions for health care workers was essential, and learning points from this pilot include notifying delegates in advance to bring a personal mobile telephone; a practical session on how to send SMS text messages; and expanding the live scenario workshop component.

**Table 1:** Critical success factors for SMS for Life project implementation

Factor	Comments
Inclusion in government mainstream programmes	Ensures that the system becomes mandatory and included in job descriptions/accountability of district personnel
	Project tasks are not dependent on external resources

Fixed timescale	A specific time period for implementation is advisable
	A period of 12-18 months is recommended
	Strict timelines and strong project management are essential

Mobile telephone coverage	Mobile telephone coverage within at least 2-3 hours of the health facility is mandatory for project participation
	Future implementation should be focused on areas with adequate coverage

Free mobile telephone response number	Personal telephones frequently have no credit Free number ensures that cost is not a deterrent to sending stock count replies

Use of personal mobile telephones	Avoids problems of maintenance, familiarity and issue of ownership associated with project-owned telephones
	Registration/deregistration permits changes to health facility staff and personal mobile telephone numbers

Airtime credit incentive for punctual stock count responses	Transmit a small amount of airtime credit to personal mobile telephones for each timely response (recommended for at least a one-year period)

Mobile telephone access to the system	Provide a mobile telephone version of the system, and Blackberry or similar devices if necessary, to permit management staff to connect to the system if computer-based access is problematic

Effective training session for health facility workers	Invitations to stress the importance of bringing a personal mobile telephone with known network coverage in the health facility area
	Include session on 'how to text'
	Expand practical component to run 5 live scenarios twice

Improved health facility store rooms	Pharmacy best practice would be assisted by standardized provision of store room equipment/shelving

Include stock counts at Zonal Stores	Weekly stock counts from Zonal Stores would provide comprehensive visibility of stock levels and stock coverage for the entire country

Other uses of cell phones and SMS texts to improve health care delivery have previously been explored in resource-constrained settings in Africa [7-10]. These have typically focused on improving patient adherence to treatment for HIV/AIDS or tuberculosis, and enhancing communication between healthcare workers and remotely-located patients [7-9]. One innovative pilot study in Zambia has used weekly SMS reports of new cases of malaria from rural health centers to provide punctual detection of positive diagnoses and thus facilitate timely intervention to prevent an upsurge in transmission [10]. Such approaches have proved technically feasible and achieved good outcomes, such that mobile phone-based systems appear likely to expand as part of rural health care provision in Africa. The current study, which to our knowledge is the first to apply an SMS-centered system to manage stock levels at a local level, has demonstrated another practical and successful application of the technology. As use of RDTs expands in Tanzania, ACT stock levels would be reduced accordingly and tight management of stocking would become even more critical to avoid stockouts. The current system would then become even more valuable - and additionally offer weekly monitoring of RDTs supplies to avoid reversion to clinically-based diagnosis for which ACT stocks then be inadequate to treat.

In conclusion, this innovative pilot shows that the SMS for Life system has the potential to alleviate restricted anti-malarial drug availability in rural areas, one of the major barriers to effective management of the disease. The system is flexible, scalable and compatible with any mobile telephone network, and can be implemented in any country with minimal tailoring. Costs for implementing the system on a wider scale would be low, at approximately US$5,000 per district in Tanzania, with the largest single item being the per diem payment to health facility staff to attend training sessions. Ongoing post-implementation costs would be approximately $7,000 per district per year, including the weekly incentive payments. The system could also usefully be applied to stock management of other priority medicines in similar settings. Finally, the public-private partnership model piloted here effectively harnessed a series of diverse skills and expertise and could be utilized to tackle other societal problems.

## Abbreviations

ACT: artemisinin-based combination therapy; AL: artemether-lumefantrine; DMO: District Medical Officer; NMCP: National Malaria Control Programme; WHO: World Health Organization

## Conflicts of interests

J Barrington is an employee of Novartis Pharma. Olympia Wereko-Brobby was an intern at Novartis Pharma during her contribution to the project. The other authors have no conflicts of interest to declare.

## Authors' contributions

*Jim Barrington*, as programme director, developed the initial concept for the project, established the project team, contacted and liaised with all project partners, coordinated activity throughout, and prepared the project report upon which the manuscript is based.

*Olympia Wereko-Brobby *provided organizational support throughout the project to the program director and contributed to development of the project report.

*Peter Ward *was the project manager throughout, and in addition significantly refined the final project reports and rewrote the guidance document.

*Winfred Mwafongo *undertook all within-country organization of health facility visits and training session, and conducted the training of health facility staff for the project.

*Seif Kungulwe*, as District Medical Office Lindi Rural, contributed to training sessions for health facility staff, and was a highly active participant in all implementation aspects of the project.
